# Desmogleins 1, 3, and E-cadherin immunohistochemical expression within mucocutaneous pemphigus vulgaris

**DOI:** 10.11604/pamj.2022.42.186.35429

**Published:** 2022-07-07

**Authors:** Muhanad Lebnan Alshami, Fawaz Aswad, Bashar Abdullah

**Affiliations:** 1 Department of Dentistry, Dijlah University College, Baghdad, Iraq,; 2 Department of Oral Diagnosis, College of Dentistry, University of Baghdad, Baghdad, Iraq

**Keywords:** Pemphigus vulgaris, desmoglein1, desmoglein3, E-cadherin

## Abstract

**Introduction:**

pemphigus vulgaris (PV) is an autoimmune condition characterized by the loss of adhesion between the epithelial cells and blister formation. The production of autoantibodies against desmosomal proteins, namely, desmoglein (DSG) 1 and DSG3, is considered a main event of PV. A full understanding of the role of adhesion molecules in the pathogenesis of PV is not fully elucidated yet. This study aimed to evaluate and correlate the immunohistochemical expression of E-cadherin (E-cad), DSG1, and DSG3 proteins in oral and skin PV.

**Methods:**

this study was a retrospective analysis study. Positive PV cases were stained with anti-E-cad, anti-DSG1, and anti-DSG3 antibodies. The expression of each marker was determined by two pathologists according to an established scoring system: (E-cad: negative, weak, moderate, and strong), (DSG1: negative, weak, and strong), and (DSG3: negative and positive). The Chi-square and Pearson´s correlation tests were used to statistically analyze the data.

**Results:**

forty-three biopsies (26 skin and 17 oral tissue samples) from 22 males and 21 female PV patients were included. The median age was 40.50 years. In total, the immunohistochemical expression was negative for DSG3, E-cad, and DSG1 in 81.4%, 18.5%, and 16.4%, respectively. DSG1 expression was significantly higher in males than females. A statistically significant correlation was found between E-cad and DSG3 expressions.

**Conclusion:**

a significant difference in the expression of markers of both oral and skin PV was absent. Downregulation of DSG3 expression was the hallmark feature that also showed a positive correlation with E-cad expression.

## Introduction

Adherens junctions and desmosomes play critical roles in maintaining cell and tissue integrity by mediating strong adhesion between adjacent epithelial cells [1]. The main adhesion molecules of adherens junctions are transmembrane proteins called E-cad, which are members of the classical cadherin family [2]. Desmosomalcadherins are the main adhesion proteins within desmosomes, which are made up of four desmogleins (DSG) (DSG1 to DSG4) and three desmocollins (DSC) (DSC1 to DSC3) encoded by different genes [3]. The expression of the desmosomalcadherins within epithelial layers is not even. Whereas DSG1 is found in all layers of the skin, DSG3 is only found in the deep epidermis in two or three layers. Additionally, DSG3 is found in higher concentrations in mucous membranes such as the oral epithelium than DSG1, which is only found in trace amounts [4]. The cytoplasmic parts of E-cad and desmosomal cadherin are linked to the actin cytoskeleton and intermediate filaments, respectively [5,6]. Intercellular connection formation is a dynamic process. In brief, the interaction of E-cads, in conjunction with actin polymerization and rearrangement, coordinates and regulates downstream responsible for the synthesis of desmosomes that anchor intermediate filaments. Subsequently, desmosomes develop the ability to maintain cell-cell attachment following maturation. The damage of desmosomes and adherens junctions can result in a process in which epithelial cell-cell adhesion is disrupted, which is known as acantholysis [7].

Pemphigus vulgaris (PV) is a life-threatening condition that is characterized by keratinocyte adhesion disruption and blister formation [8]. Worldwide, PV incidence varies from 0.07 to 1.6 per 100,000 people [9]. Females have more PV lesions than males, and the average age ranges between 40 and 60 years [10,11]. Pemphigus vulgaris lesions develop in the mucous membrane and may involve the skin [12]. Pemphigus vulgaris clinical manifestations include the formation of blisters filled with clear fluid or, in some cases, pus or blood. The blisters have a high proclivity to rupture within 24 hours of the eruption, resulting in an ulcer or erosion lesion associated with bleeding and oozing [13]. The microscopic image of an intact PV blister shows a cleft within the epithelia just above the basal cell layer. The cells of the spinous layer exhibit acantholysis and appear as round cells, which are referred to as tzanck cells. The epithelium could contain clear fluid, a small number of inflammatory cells, and a few epithelial sheets, all of which would occupy the available space. The underlying connective tissue demonstrates an inflammatory response which includes an increase in vascularity and inflammatory cells; the severity of the inflammatory response generally ranges from mild to moderate [14,15].

The epithelial break and blister formation in PV is mediated by autoantibodies that target the adhesion molecules between the epithelial cells. IgG4 has been documented as a pathogenic factor in the acute phase of the disease, whereas the remission phase is linked to IgG1. Other autoantibodies also play a role in PV pathogenesis, such as IgM and IgE [16]. The main targets for the autoantibodies are the desmosome junctions' proteins, especially the DSG3 (mucosal type) or DSG3 and DSG1 (mucocutaneous type) [17]. The mechanism of acantholysis is not confirmed yet. However, E-cad is found to be important in desmosome assembly, so it is believed that these findings are clinically significant because they highlight E-cad as a target of pemphigus autoantibodies [18].

The aims of the present study are to evaluate and correlate the immunohistochemical (IHC) expression of DSG1, DSG3, and E-cad in oral and skin PV tissue samples.

## Methods

**Study design:** this retrospective analysis was conducted on paraffin-embedded tissue blocks with associated histopathological examination reports for forty-three confirmed PV cases between 2019 and 2021.

**Ethical approval:** this study was conducted after obtaining ethical approval from the Ethics Committee, College of Dentistry, University of Baghdad (Ref # 297, date: 01/04/2021).

**Study samples:** the samples were retrieved from the Histopathological Laboratory, Teaching Hospital, Ministry of Health and Oral Pathology Laboratory, College of Dentistry, University of Baghdad. Age, sex, and whether the biopsy was taken from the skin or the mouth were taken from the relevant reports for each case.

**Samples processing:** five sections with a 4 µm thickness were taken from each block; the sections were loaded on microscopic charge slides. One slide for each case was stained with hematoxylin and eosin (H&E) to reevaluate the histopathological picture of PV and ensure that the tissue was adequate. The remaining four slides were used for IHC analysis.

**Immunohistochemistry:** the IHC process involves dealing with the slides through several sequential steps, which include deparaffinization, rehydration, antigen retrieval, blocking the endogenous peroxidase, and then the step of adding primary and secondary antibodies. The primary antibodies used in this study were anti-E-cad (Ref: sc-8426-1: 50 dilution), anti-DSG1 (Ref: sc-137164-1: 50 dilution), and anti-DSG3 (Ref: sc-53487-1: 50 dilution), all purchased from Santa Cruz Biotechnology, Santa Cruz, California, USA. Finally, the slides were treated with diaminobenzidine [19] reagents and Mayer's hematoxylin. The prepared slides were dehydrated. In the current study, negative and positive controls were used. The positive control for anti-E-cad, anti-DSG1, and anti-DSG3 antibodies was normal gingiva that was taken during treatment of the gummy smile.

**Scoring system:** the assessment of IHC expression was done by observing five random fields in each section by two pathologists using a light microscope at power 40 X. The expression of E-cad was assessed using a semiquantitative method based on the staining intensity: no staining (0), weak staining (1), moderate staining (2), and strong staining [20], and the percentage of cells stained positively was as follows: no cells stained positively (0), less than 10% stained cells (1), 10% to 29% stained cells (2), 30% to 59% stained cells [20], and >60% stained cells (4). The scores of both intensity and percentage were multiplied to obtain the final E-cad assessment scores: negative (0-1); mild (2-3); moderate (4-8); and strong (9-12) [21]. The scoring system for DSG3 was semiquantitative and included positive cell percentage: no stained cells (0), 1% to 50% stained cells (1), and 51% to 100% stained cells (2); and the intensity of staining: no staining (0), weak staining (1), moderate staining (2), and strong staining [20]. The final evaluations of DSG3 were based on a summation of scores for both intensity and percentage of positive staining cells. If the results ranged between 0 to 3, these were considered negative, but if the results were equal to 4 or 5, these were considered positive [22]. The quantitative method was applied to DSG1 scoring, (0: negative) fewer than 5% stained cells, (1: weak stain) 5% to 50% stained cells, and (2: strong stain) greater than 50% stained cells [23].

**Statistical analysis:** Statistical Package for the Social Sciences (SPSS) version 16.0 was used for all statistical analyses. The characteristics of the patients were presented descriptively. The Chi-square test determined the relationship between antibodies expression and the variables. The Spearman's correlation test was used to determine the correlations between DSG1, DSG3, and E-cad. A p-value <0.05 was used to determine the significance level.

## Results

Immunohistochemical staining for DSG1, DSG3, and E-cad was performed on 43 PV samples (males 51.2% and females 48. 8%). The age of patients ranged from 17 to 67, with an average age of 39.5 years (male 38.41 years and female 40.68 years). The patients were classified into two groups according to age standards of World Health Organization (WHO): 44 years or below and above 44 years [24]. The samples were taken from 26 skin samples and 17 oral cavity samples. The demographic information is shown in Table 1.

**Table 1:** demographic variables of the study population

Demographic variables			
Age (years)	Mean ± SD	Median	Min-max
Male	38.41 ± 12.69	38.50	17 - 67
Female	40.68 ± 10.43	41.50	17 - 60
Total	39.55 ± 11.53	40.50	17 - 67
**Age groups (frequency, %)**			
17 to 44	27, 62.8		
>44	16, 37.2		
**Sex (frequency, %)**			
Male	22, 51.2		
Female	21, 48.8		
**Site (frequency, %)**			
Skin	26, 60.5		
Oral	17, 39.5		
Total	43, 100		

**Figure 1:**
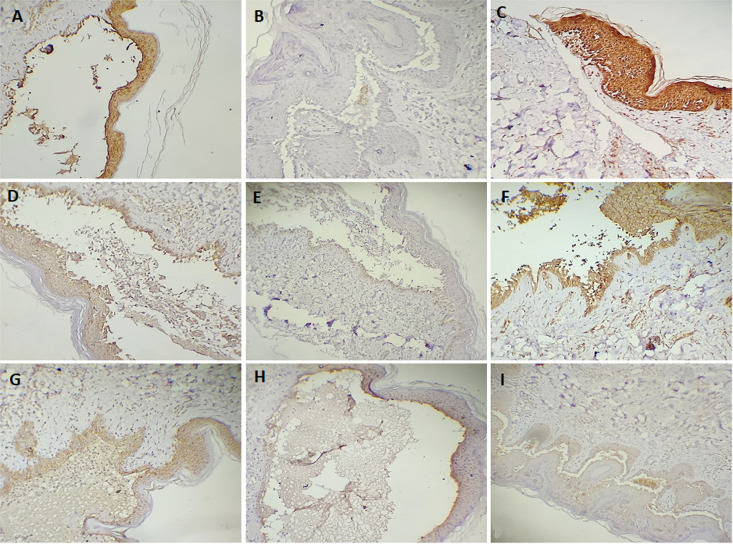
the expression of desmoglein 1 (DSG1), desmoglein 3 (DSG3), and E-cadherin (E-cad) in multiple pemphigus vulgaris cases: A) positive DSG3 expression; B) negative DSG3 expression; C) strong positive DSG1 expression; D) weak positive DSG1 expression; E) negative DSG1 expression; F) strong E-cad expression; G) moderate E-cad expression; H) mild E-cad expression; and I) negative E-cad expression

The microscopic examination of immunohistochemical expression (Figure 1) revealed that E-cad expressions were negative in 18.6% samples and positive in 79.5% samples. Positive samples include 14 with mild staining, 14 with moderate staining, and 7 with strong staining. There was no correlation between E-cad and demographic variables. DSG3 expression was negative in 81.4% samples and positive in 18.7% samples (Figure 1). The correlation between the DSG3 expressions and the demographic variables is not significant. The positive DSG1 samples were 83.7%, with 60.5% being strong positive and 23.2% samples being weak positive. Negative DSG1 staining was found in 16.3% samples (Figure 1). Males had higher DSG1 expression since 80% of them had strong staining. All immunohistochemical expression scores of the included markers and the correlations with variables are illustrated in Table 2. All expression in PV cases compared with positive controls that expressed strong stain for DSG1, DSG3, and E-cad. DSG1 expression was non-significantly associated with other demographic variables. Figure 2 illustrates the correlations between the expression of markers in this study. Correlations between E-cad and DSG3 expression were significant (p value=0.003), whereas correlations between DSG1 and E-cad and DSG3 expressions were not significant.

**Table 2:** expression of cellular adhesion markers according to demographic variables

Demographic variables	Immunohistochemical expression scores #								
	E-cad				DSG3		DSG1		
	Negative	Mild	Moderate	Strong	Negative	Positive	Negative	Weak	Strong
**Age groups**									
19 to 44	6, 22.2	4, 14.8	14, 51.9	3, 11.1	24, 88.9	3, 11.1	4, 14.8	6, 22.2	17, 63.0
> 44	3, 18.8	6, 37.5	4, 25.0	3, 18.8	12, 75.0	4, 25.0	3, 18.8	4, 25.0	9, 56.3
p value	0.218				0.394		0.903		
**Sex**									
Male	5, 22.7	7, 31.8	8, 36.4	2, 9.1	19, 86.4	3, 13.6	1, 4.5	3, 13.6	18, 81.8
Female	4, 19.0	3, 14.3	10, 47.6	4, 19.0	17, 81.0	4, 19.0	6, 28.6	7, 33.3	8, 38.1
p value	0.461				0.698		0.011*		
**Site**									
Skin	4, 15.4	7, 26.9	12, 46.2	3, 11.5	20, 76.9	6, 23.1	6, 23.1	5, 19.2	15, 57.7
Oral	5, 29.4	3, 17.6	6, 35.3	3, 17.6	16, 94.1	1, 5.9	1, 5.9	5, 29.4	11, 64.7
p value	0.591				0.215		0.300		
Sub-total	9, 20.9	10, 23.3	18, 41.9	6, 14.0	36, 83.7	7, 16.3	7, 16.3	10, 23.3	26, 60.5
Total	43, 100				43, 100		43, 100		

# Frequency, %; * significant difference at p<0.05 by Chi-square test

**Figure 2:**
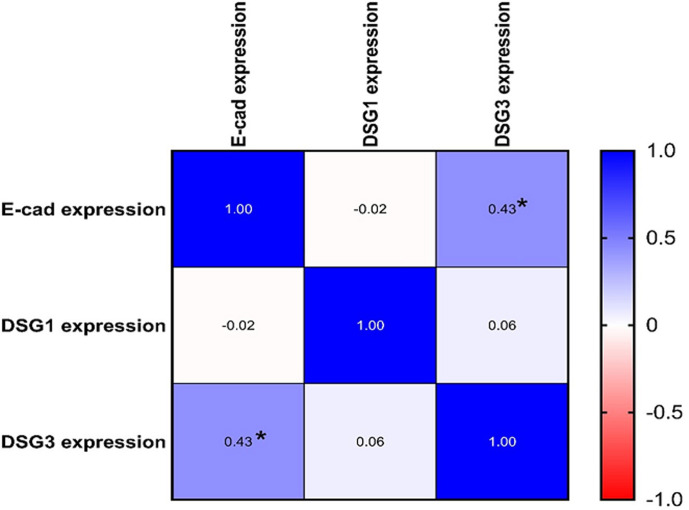
correlation of cellular adhesion molecules, E-cadherin (E-cad) is positively and significantly associated with desmoglein (DSG) 3 but not with DSG1; in addition, DSG1 and DSG3 did not show any significant correlation (*p-value = 0.003 by Spearman’s correlation test)

## Discussion

Pemphigus vulgaris (PV) patients were found to be nearly 40 years old on average in this study. Previous epidemiological studies have revealed a different range of average ages. Studies in Iran [25], China [26], and Brazil [27] indicated that the average age was between 41 and 44 years, whereas studies in Turkey [28], Poland [29], Croatia [30], and Japan [31] have suggested that the average age was between 50 and 55 years. The variation in results could be due to a difference in study sample size and ethnic variations.

More than two-thirds of the study samples showed negative DSG3 expression. Although there was no significant correlation between the DSG3 expression and the variables, almost all the oral PV and two-thirds of the skin PV were DSG3-negative. More than half of the total cases showed disruption in DSG1 expression. This result agreed with previous studies that mentioned that DSG3 and DSG1 were the main targets for PV autoantibodies, mainly in cutaneous PV that needed both desmogleins to be attacked by the autoantibodies to develop [32,33]. According to sex, 80% of females and also 80% of males showed disruption in DSG3 expression, while more than 60% of females showed disruption in DSG1 expressions, and strong DSG1 stain appeared in more than 80% of males. Many previous studies have shown that females are more susceptible to severe PV than males and showed cutaneous involvement [34]. E-cad is the core of adherence junctions within epithelial cell layers. The downregulation of E-cad expression could lead to acantholysis between epithelial cells and blister formation, especially when there is a disruption in desmosomal junctions [35]. E-cad expressions' interruption was seen in more than two-thirds of studied samples, however, E-cad expression showed no significant difference among the variables. This outcome is in accordance with many researches that found PV patients' serum demonstrated autoantibodies against E-cad proteins and in another study. In addition, E-cad has been detected in over two-thirds of 80 PV patients' sera using enzyme-linked immunosorbent assay (ELISA) and immunoprecipitation with western blotting [36]. The E-cad homology with DSG1 and DSG3 could enhance cross-reactivity reaction of these cellular junction molecules. In other words, the antibodies that target the DSG1 and DSG3 can target the E-cad and vice versa [37,38].

The correlations between markers´ immunohistochemical expression in the present study showed a significant correlation between the E-cad and DSG3 expressions. This result comes with the outcome of previous studies that stated that non-DSG targets play a role in PV pathogenesis. The interaction between cadherin proteins (E-cad and DSG3) is a bi-directional causing disruptions in both molecules [7].

The present study followed a retrospective design depending on archived samples from PV patients, the absence of severity measurements and follow-up are the main limitations of this study. Large study samples are recommended for future studies.

## Conclusion

A significant difference in the expression of markers of both oral and skin PV was absent. Downregulation of DSG3 expression was the hallmark feature that also showed a positive correlation with E-cad expression.

### What is known about this topic

- *Pemphigus vulgaris (PV) is an autoimmune condition that leads to epithelial cell acaantholysis;*
- *the main targets for the autoantobius are DSG3 and DSG1*.

### What this study adds

- *Pemphigus vulgaris (PV) is multifactorial condition;*
- *E-cad could be involved in the PV pathogenies;*
- *there was interaction between DSG3 and E-cad*.

## References

[1] Wan H, Gadmor H, Brown L (2018). Anchoring junctions in the oral mucosa: adherens junctions and desmosomes. Oral Mucosa in Health and Disease: Springer.

[2] Coopman P, Djiane A (2016). Adherens junction and E-cadherin complex regulation by epithelial polarity. Cell Mol Life Sci.

[3] Broussard JA, Getsios S, Green KJ (2015). Desmosome regulation and signaling in disease. Cell Tissue Res.

[4] Spindler V, Drenckhahn D, Zillikens D, Waschke J (2007). Pemphigus IgG causes skin splitting in the presence of both desmoglein 1 and desmoglein 3. Am J Pathol.

[5] Lecuit T, Yap AS (2015). E-cadherin junctions as active mechanical integrators in tissue dynamics. Nat Cell Biol.

[6] Kang H, Weiss TM, Bang I, Weis WI, Choi HJ (2016). Structure of the intermediate filament-binding region of desmoplakin. PLoS One.

[7] Tsang SM, Brown L, Lin K, Liu L, Piper K, O'Toole EA (2012). Non-junctional human desmoglein 3 acts as an upstream regulator of Src in E-cadherin adhesion, a pathway possibly involved in the pathogenesis of pemphigus vulgaris. J Pathol.

[8] Huang Y, Jedličková H, Cai Y, Rehman A, Gammon L, Ahmad US (2021). Oxidative stress-mediated YAP dysregulation contributes to the pathogenesis of pemphigus vulgaris. Front Immunol.

[9] Ferreira Costa MT, Oyafuso LKM, Gamba MA, Woo KY (2021). The nursing assessment of pemphigus vulgaris ulcers. World Council of Enterostomal Therapists Journal.

[10] Kridin K, Zelber-Sagi S, Bergman R (2017). Pemphigus vulgaris and pemphigus foliaceus: differences in epidemiology and mortality. Acta Derm Venereol.

[11] Kayani M, Aslam AM (2017). Bullous pemphigoid and pemphigus vulgaris. BMJ.

[12] Melchionda V, Harman K (2019). Pemphigus vulgaris and pemphigus foliaceus: an overview of the clinical presentation, investigations and management. Clin Exp Dermatol.

[13] Di Lernia V, Casanova DM, Goldust M, Ricci C, conceptual (2020). Pemphigus vulgaris and bullous pemphigoid: update on diagnosis and treatment. Dermatol Pract Concept.

[14] Manocha A, Tirumalae R (2021). Histopathology of pemphigus vulgaris revisited. Am J Dermatopathol.

[15] Hrabovska Z, Jautova J, Hrabovsky V (2017). A study of clinical, histopathological and direct immunofluorescence diagnosis in pemphigus group utility of direct immunofluorescence. Bratisl Lek Listy.

[16] Popescu IA, Statescu L, Vata D, Porumb-Andrese E, Patrascu AI, Grajdeanu I-A (2019). Pemphigus vulgaris-approach and management. Exp Ther Med.

[17] Porro AM, Seque CA, Ferreira MCC, Enokihara MMS (2019). Pemphigus vulgaris. An Bras Dermatol.

[18] Amber KT, Valdebran M, Grando SA (2018). Non-desmoglein antibodies in patients with pemphigus vulgaris. Front Immunol.

[19] Kaomongkolgit R, Sarideechaigul W, Klanrit P, Phothipakdee P (2021). Clinical profile of oral pemphigus vulgaris in Thai patients. Family Medicine Primary Care Review.

[20] Riley JL, Gilbert GH (2001). Orofacial pain symptoms: an interaction between age and sex. Pain.

[21] Fedchenko N, Reifenrath J (2014). Different approaches for interpretation and reporting of immunohistochemistry analysis results in the bone tissue - a review. Diagn Pathol.

[22] Huang CC, Lee TJ, Chang PH, Lee YS, Chuang CC, Jhang YJ (2010). Desmoglein 3 is overexpressed in inverted papilloma and squamous cell carcinoma of sinonasal cavity. Laryngoscope.

[23] Wong MP, Cheang M, Yorida E, Coldman A, Gilks CB, Huntsman D (2008). Loss of desmoglein 1 expression associated with worse prognosis in head and neck squamous cell carcinoma patients. Pathology.

[24] Dyussenbayev A (2017). Age periods of human life. Advances in Social Sciences Research Journal.

[25] Asilian A, Yoosefi A, Faghini G (2006). Pemphigus vulgaris in Iran: epidemiology and clinical profile. Skinmed.

[26] Zhu X, Pan J, Yu Z, Wang Y, Cai L, Zheng S (2014). Epidemiology of pemphigus vulgaris in the Northeast China: a 10-year retrospective study. J Dermatol.

[27] Gonçalves GAP, Brito MMC, Salathiel AM, Ferraz TS, Alves D, Roselino AMF (2011). Incidence of pemphigus vulgaris exceeds that of pemphigus foliaceus in a region where pemphigus foliaceus is endemic: analysis of a 21-year historical series. An Bras Dermatol.

[28] Askin O, Ozkoca D, Kutlubay Z, Mat MC (2020). A retrospective analysis of pemphigus vulgaris patients: demographics, diagnosis, co-morbid diseases and treatment modalities used. North Clin Istanb.

[29] Serwin AB, Koper M, Flisiak I (2018). Incidence of pemphigus vulgaris and pemphigus foliaceus in North-East Poland (Podlaskie Province)-a 15-year (2001-2015) bicentric retrospective study. Int J Dermatol.

[30] Ljubojevic S, Lipozencic J, Brenner S, Budimcic D (2002). Pemphigus vulgaris: a review of treatment over a 19-year period. J Eur Acad Dermatol Venereol.

[31] Ishii N, Maeyama Y, Karashima T, Nakama T, Kusuhara M, Yasumoto S (2008). A clinical study of patients with pemphigus vulgaris and pemphigus foliaceous: an 11-year retrospective study (1996-2006). Clin Exp Dermatol.

[32] Ruocco V, Ruocco E, Schiavo AL, Brunetti G, Guerrera LP, Wolf R (2013). Pemphigus: etiology, pathogenesis, and inducing or triggering factors: facts and controversies. Clin Dermatol.

[33] Pollmann R, Schmidt T, Eming R, Hertl M (2018). Pemphigus: a comprehensive review on pathogenesis, clinical presentation and novel therapeutic approaches. Clin Rev Allergy Immunol.

[34] Svecova D (2015). Pemphigus vulgaris: a clinical study of 44 cases over a 20-year period. Int J Dermatol.

[35] Rötzer V, Hartlieb E, Vielmuth F, Gliem M, Spindler V, Waschke J (2015). E-cadherin and Src associate with extradesmosomal Dsg3 and modulate desmosome assembly and adhesion. Cell Mol Life Sci.

[36] Oliveira ME, Culton DA, Prisayanh P, Qaqish BF, Diaz LA (2013). E-cadherin autoantibody profile in patients with pemphigus vulgaris. Br J Dermatol.

[37] Sinha AA, Sajda T (2018). The evolving story of autoantibodies in pemphigus vulgaris: development of the “super compensation hypothesis”. Front Med (Lausanne).

[38] Völlner F, Ali J, Kurrle N, Exner Y, Eming R, Hertl M (2016). Loss of flotillin expression results in weakened desmosomal adhesion and Pemphigus vulgaris-like localisation of desmoglein-3 in human keratinocytes. Sci Rep.

